# Supplementary material for: Federated Knowledge Retrieval Elevates Large Language Model Performance on Biomedical Benchmarks
Source: bioRxiv. 2025 Aug 2:2025.08.01.668022. Preprint. [Version 1] doi: 10.1101/2025.08.01.668022 (PMC12324469; doi:10.1101/2025.08.01.668022)
Supplement: 1 — Supplementary Figure S1: Detailed pipeline for BTE-RAG Supplementary Figure S2: Performance of BTE-RAG versus an LLM-only baseline on the gene-centric benchmark using gpt-4o-mini. Supplementary Figure S3: Performance of BTE-RAG versus an LLM-only baseline on the gene-centric benchmark using gpt-4o. Supplementary Figure S4: Cosine-similarity profile for the metabolite-centric benchmark using GPT-4o-mini in LLM-only mode. Supplementary Figure S5: Distribution of answer similarities for the metabolite-centric benchmark using GPT-4o-mini in BTE-RAG mode. Supplementary Figure S6: Distribution of answer similarities for the metabolite-centric benchmark using GPT-4o in BTE-RAG mode. Supplementary Figure S7: Rank-ordered cosine similarities between model predictions and ground-truth answers on the metabolite-centric benchmark, across context filtering thresholds. Supplementary Figure S8: Cosine-similarity profile for the drug-centric benchmark using GPT-4o-mini in LLM-only mode. Supplementary Figure S9: Distribution of answer similarities for the drug-centric benchmark using GPT-4o-mini in BTE-RAG mode. Supplementary Figure S10: Distribution of answer similarities for the drug-centric benchmark using GPT-4o in BTE-RAG mode. Supplementary Figure S11: Rank-ordered cosine similarities between model predictions and ground-truth answers on the drug-centric benchmark, across context filtering thresholds. Supplementary Table S1: System prompts used for each task and model [file NIHPP2025.08.01.668022V1-supplement-1.pdf]

bioRxiv preprint doi: https://doi.org/10.1101/2025.08.01.668022; this version posted August 2, 2025. The copyright holder for this preprint (which was not certified by peer review) is the author/funder, who has granted bioRxiv a license to display the preprint in perpetuity. It is made available under aCC-BY 4.0 International license.

Supplementary Figures

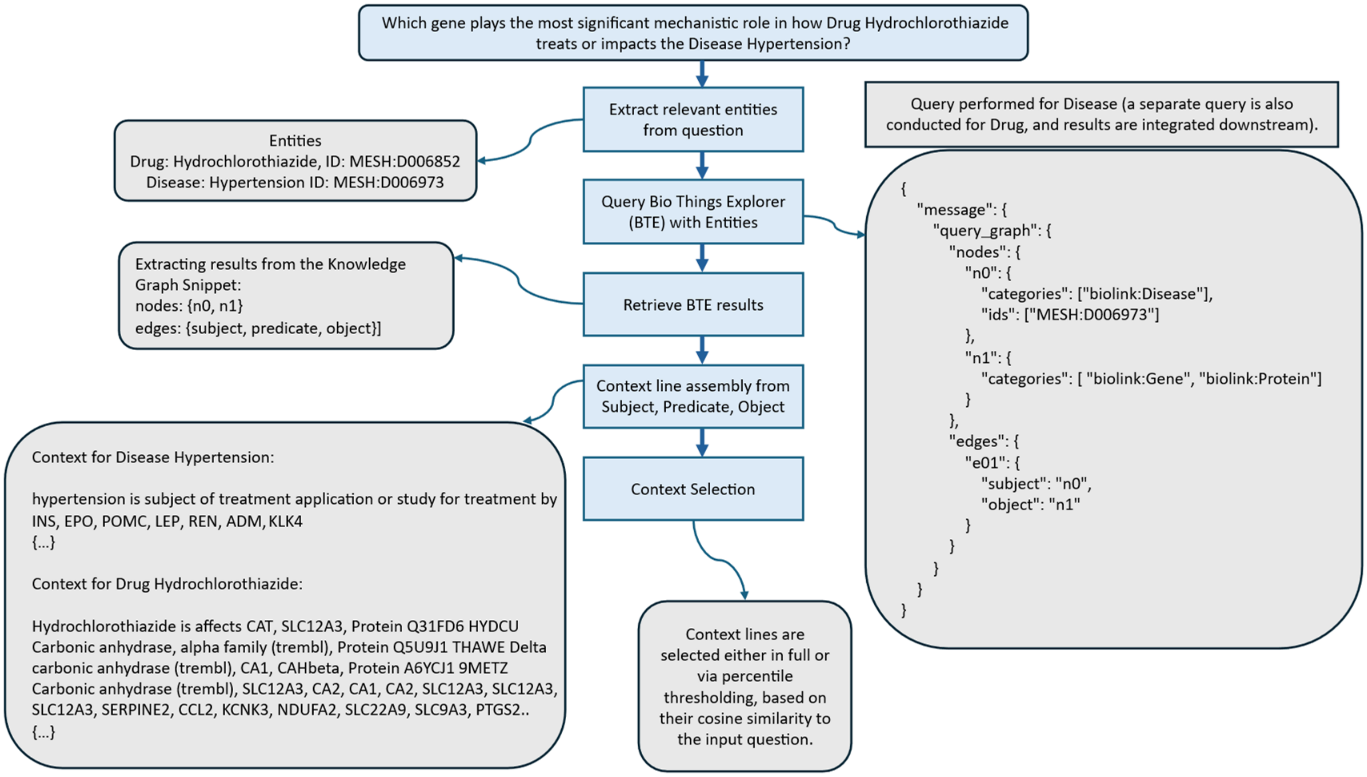

Figure S1: Detailed pipeline for BTE-RAG

Supplementary Figure S1 depicts the end-to-end workflow through which the BTE-RAG retrieval module converts a biomedical question into the evidence snippets ultimately supplied to the language-model reasoner. Beginning with an example query, "Which gene plays the most significant mechanistic role in how the drug *hydrochlorothiazide* treats or impacts the disease *hypertension*?", the system first performs named-entity recognition, normalizing the detected concepts to controlled identifiers (Drug: MESH:D006852; Disease: MESH:D006973). Each entity is then submitted to BioThings Explorer (BTE) as part of a query graph that requests mechanistically relevant genes and proteins; independent queries are executed for the drug and for the disease. BTE returns knowledge-graph sub-graphs whose nodes and edges represent subject-predicate-object triples grounded in the biomedical literature. These triples are linearized into plain-text sentences, yielding two preliminary corpora (one for the disease, one for the drug) that list, for example, genes such as *INS*, *REN*, *SLC12A3* and *PTGS2* with their associated predicates. Finally, the complete set of sentences or a percentile-filtered subset is ranked by cosine similarity to the original question, and the highest-scoring lines are selected as the "retrieved context" passed forward for answer generation.

bioRxiv preprint doi: https://doi.org/10.1101/2025.08.01.668022; this version posted August 2, 2025. The copyright holder for this preprint (which was not certified by peer review) is the author/funder, who has granted bioRxiv a license to display the preprint in perpetuity. It is made available under aCC-BY 4.0 International license.

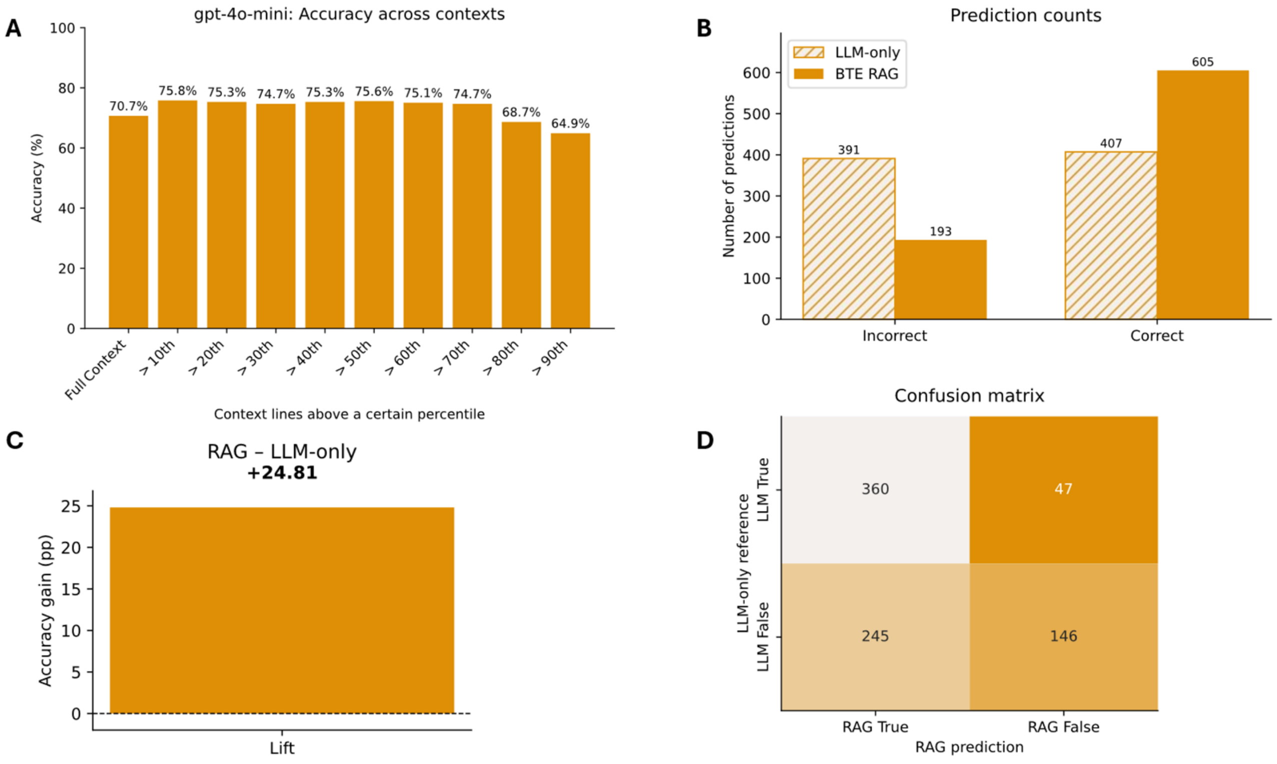

Figure S2: Performance of BTE-RAG versus an LLM-only baseline on the gene-centric benchmark using gpt-4o-mini.

(A) Overall accuracy as a function of how much of the retrieved context is retained. Bars show accuracy when only context lines above a given cosine-similarity percentile are supplied to the model (10 th–90 th) as well as when the full context is used.
(B) Breakdown of prediction counts for the 798 benchmark questions. The hatched bars represent the LLM-only condition; solid bars represent BTE-RAG.
(C) BTE-RAG outperforms the LLM-only run by +24.8 percentage points, confirming that targeted knowledge-graph snippets materially improve answer quality.
(D) Confusion matrix comparing the two methods. The upper-left cell (360 cases) denotes questions both methods answer correctly; the lower-left cell (245) highlights errors that BTE-RAG fixes; the upper-right cell (47) shows instances where retrieval introduces an error; and the lower-right cell (146) comprises questions neither approach resolves.

bioRxiv preprint doi: https://doi.org/10.1101/2025.08.01.668022; this version posted August 2, 2025. The copyright holder for this preprint (which was not certified by peer review) is the author/funder, who has granted bioRxiv a license to display the preprint in perpetuity. It is made available under aCC-BY 4.0 International license.

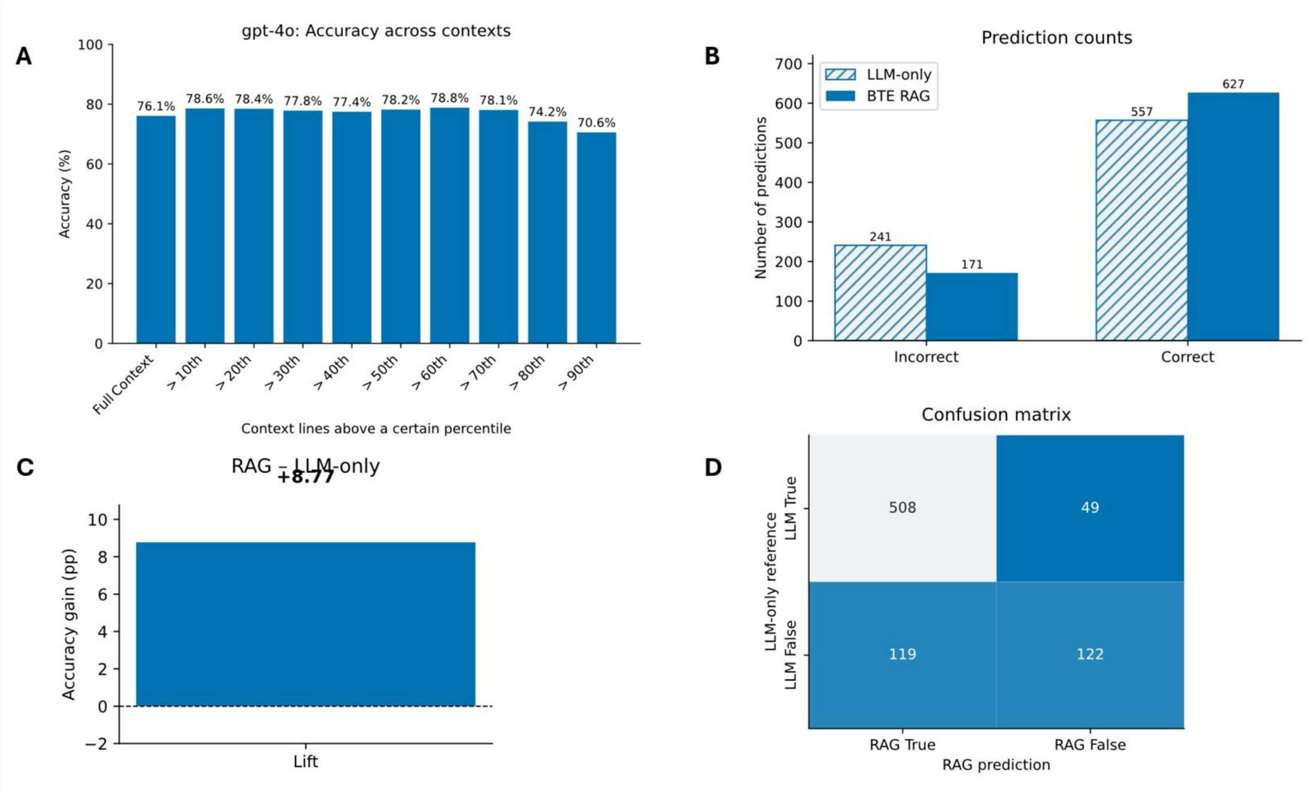

Figure S3: Performance of BTE-RAG versus an LLM-only baseline on the gene-centric benchmark using gpt-4o.

(A) Overall accuracy as a function of how much of the retrieved context is retained. Bars show accuracy when only context lines above a given cosine-similarity percentile are supplied to the model (10 th–90 th) as well as when the full context is used.
(B) Breakdown of prediction counts for the 798 benchmark questions. The hatched bars represent the LLM-only condition; solid bars represent BTE-RAG.
(C) BTE-RAG outperforms the LLM-only run by +8.8 percentage points, confirming that targeted knowledge-graph snippets materially improve answer quality.
(D) Confusion matrix comparing the two methods. The upper-left cell (508 cases) denotes questions both methods answer correctly; the lower-left cell (119) highlights errors that BTE-RAG fixes; the upper-right cell (49) shows instances where retrieval introduces an error; and the lower-right cell (122) comprises questions neither approach resolves.

bioRxiv preprint doi: https://doi.org/10.1101/2025.08.01.668022; this version posted August 2, 2025. The copyright holder for this preprint (which was not certified by peer review) is the author/funder, who has granted bioRxiv a license to display the preprint in perpetuity. It is made available under aCC-BY 4.0 International license.

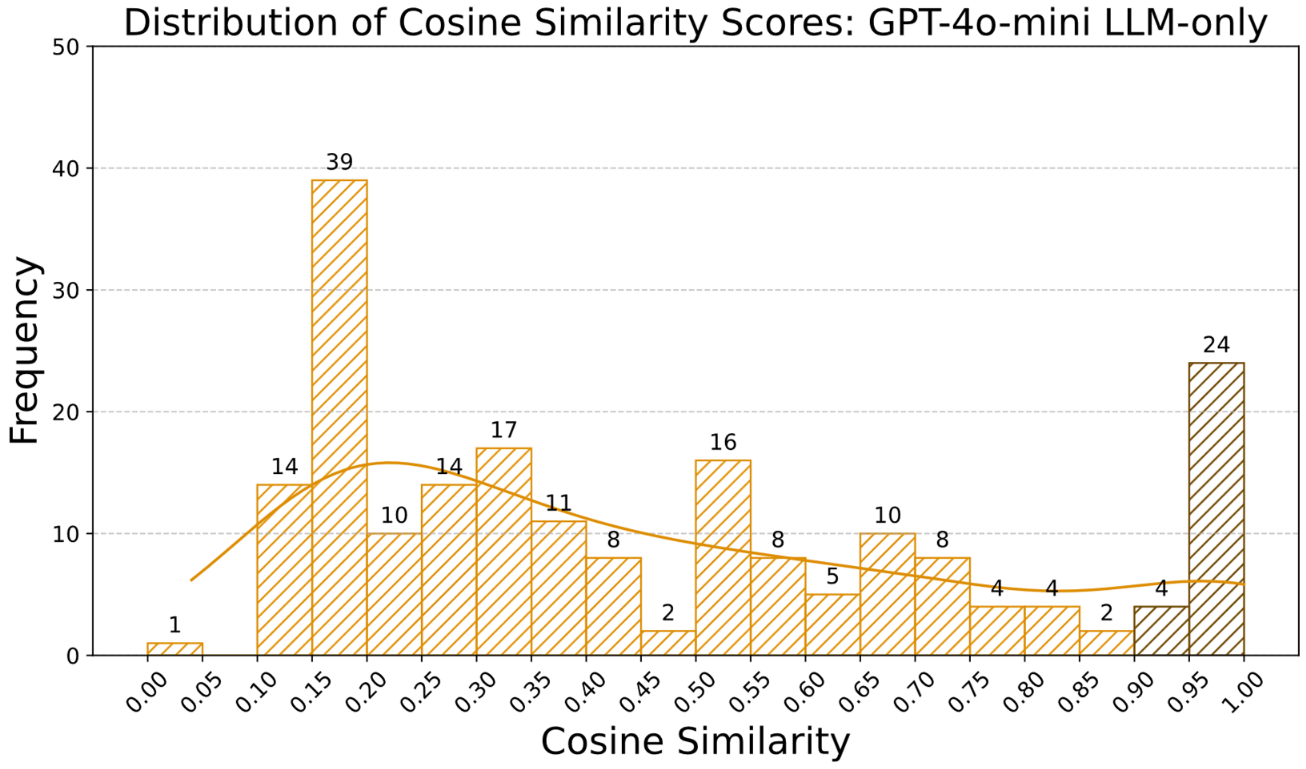

Figure S4: Cosine-similarity profile for the metabolite-centric benchmark using GPT-4o-mini in LLM-only mode.

Histogram shows the frequency of cosine-similarity scores (bin width = 0.05) between model answers and ground truth answers across 201 metabolite-related queries when using gpt-4o-mini. A smoothed kernel-density curve traces the overall score profile.

bioRxiv preprint doi: https://doi.org/10.1101/2025.08.01.668022; this version posted August 2, 2025. The copyright holder for this preprint (which was not certified by peer review) is the author/funder, who has granted bioRxiv a license to display the preprint in perpetuity. It is made available under aCC-BY 4.0 International license.

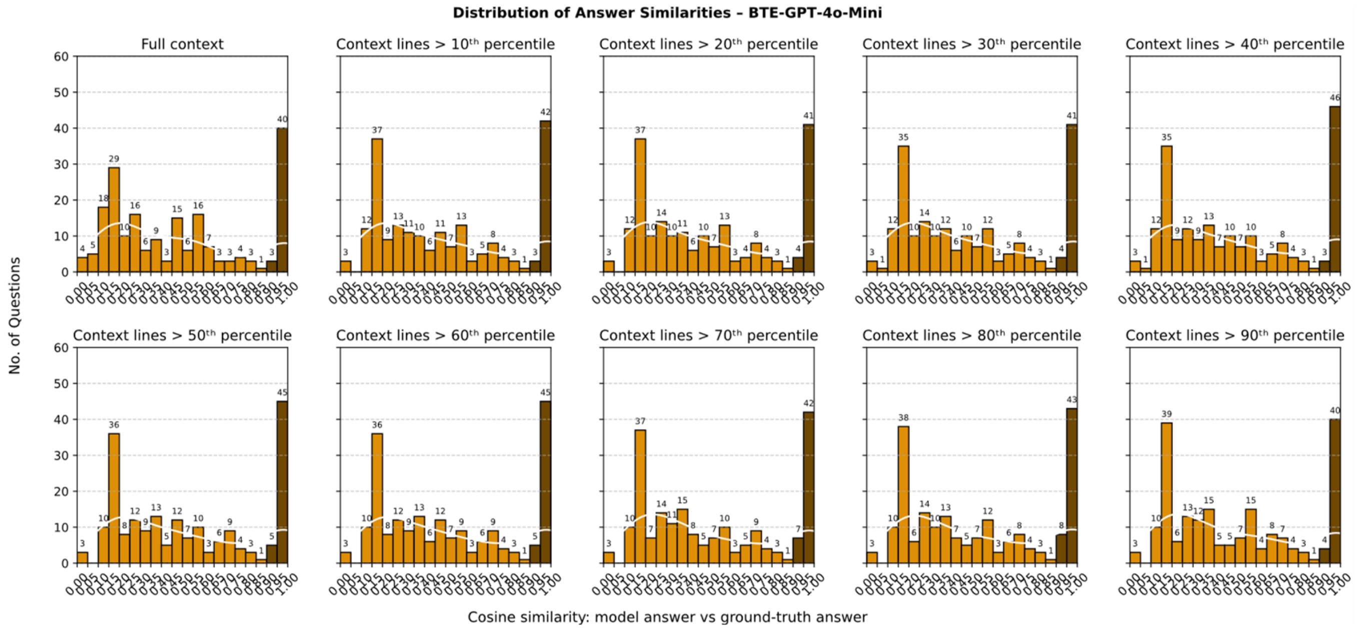

Figure S5: Distribution of answer similarities for the metabolite-centric benchmark using GPT-4o-mini in BTE-RAG mode.

Each panel shows the cosine similarity between model predictions and ground-truth answers when either the full retrieved context is used (top left) or when context lines are filtered above increasing cosine similarity percentiles (10th to 90th).

bioRxiv preprint doi: https://doi.org/10.1101/2025.08.01.668022; this version posted August 2, 2025. The copyright holder for this preprint (which was not certified by peer review) is the author/funder, who has granted bioRxiv a license to display the preprint in perpetuity. It is made available under aCC-BY 4.0 International license.

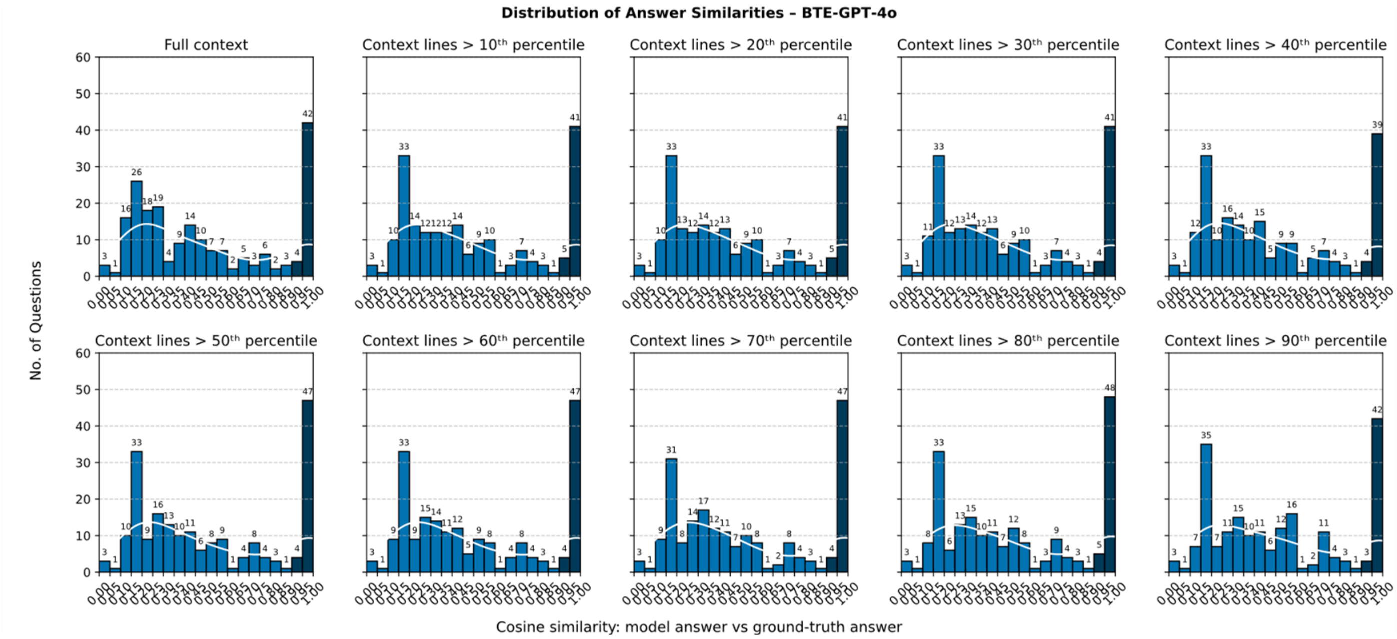

Figure S6: Distribution of answer similarities for the metabolite-centric benchmark using GPT-4o in BTE-RAG mode.

Each panel shows the cosine similarity between model predictions and ground-truth answers when either the full retrieved context is used (top left) or when context lines are filtered above increasing cosine similarity percentiles (10th to 90th).

bioRxiv preprint doi: https://doi.org/10.1101/2025.08.01.668022; this version posted August 2, 2025. The copyright holder for this preprint (which was not certified by peer review) is the author/funder, who has granted bioRxiv a license to display the preprint in perpetuity. It is made available under aCC-BY 4.0 International license.

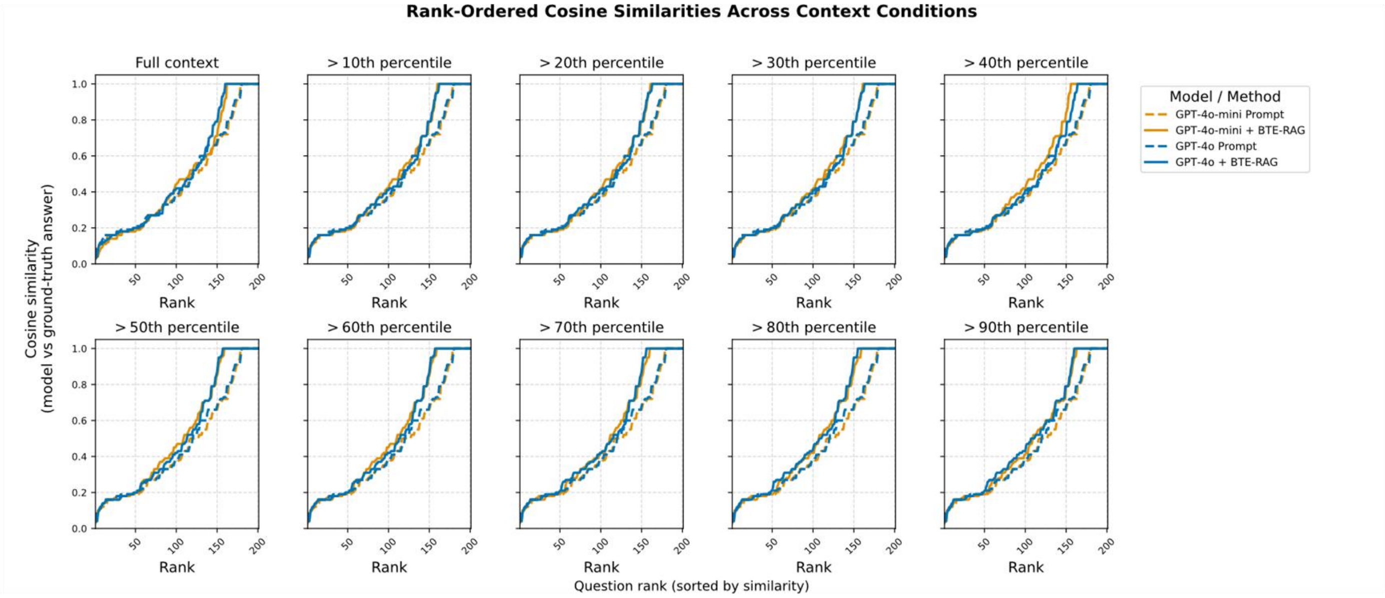

Figure S7: Rank-ordered cosine similarities between model predictions and ground-truth answers on the metabolite-centric benchmark, across context filtering thresholds.

Each panel displays results from four model–method combinations (GPT-4o-mini-Prompt (LLM-only), GPT-4o-mini + BTE-RAG, GPT-4o Prompt, GPT-4o + BTE-RAG) under either full context or filtered context lines exceeding the indicated cosine similarity percentile (10th to 90th). Question predictions are sorted by similarity, revealing how context filtering and model selection affect semantic alignment with ground truth.

bioRxiv preprint doi: https://doi.org/10.1101/2025.08.01.668022; this version posted August 2, 2025. The copyright holder for this preprint (which was not certified by peer review) is the author/funder, who has granted bioRxiv a license to display the preprint in perpetuity. It is made available under aCC-BY 4.0 International license.

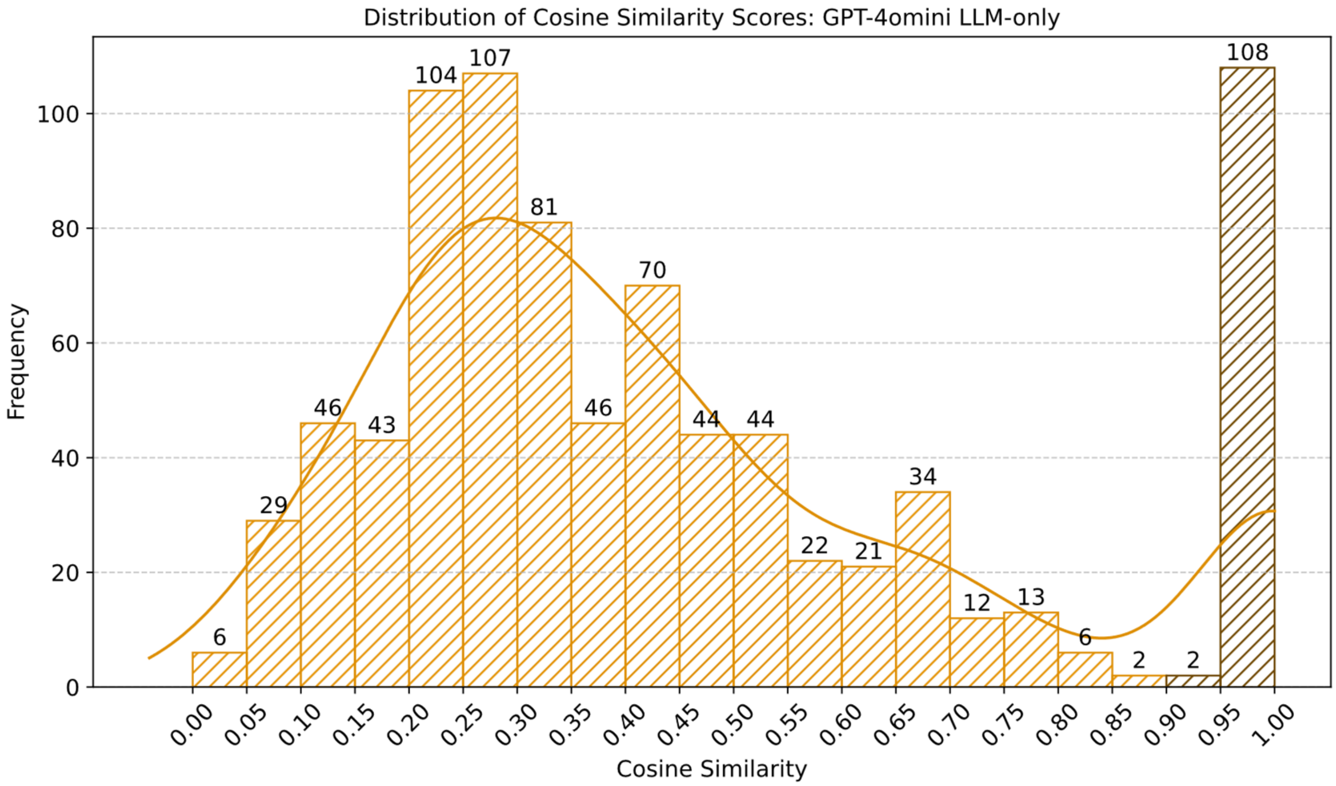

Figure S8: Cosine-similarity profile for the drug-centric benchmark using GPT-4o-mini in LLM-only mode.

Histogram shows the frequency of cosine-similarity scores (bin width = 0.05) between model answers and ground truth answers across 842 drug-biological process queries when using gpt-4o-mini. A smoothed kernel-density curve traces the overall score profile.

bioRxiv preprint doi: https://doi.org/10.1101/2025.08.01.668022; this version posted August 2, 2025. The copyright holder for this preprint (which was not certified by peer review) is the author/funder, who has granted bioRxiv a license to display the preprint in perpetuity. It is made available under aCC-BY 4.0 International license.

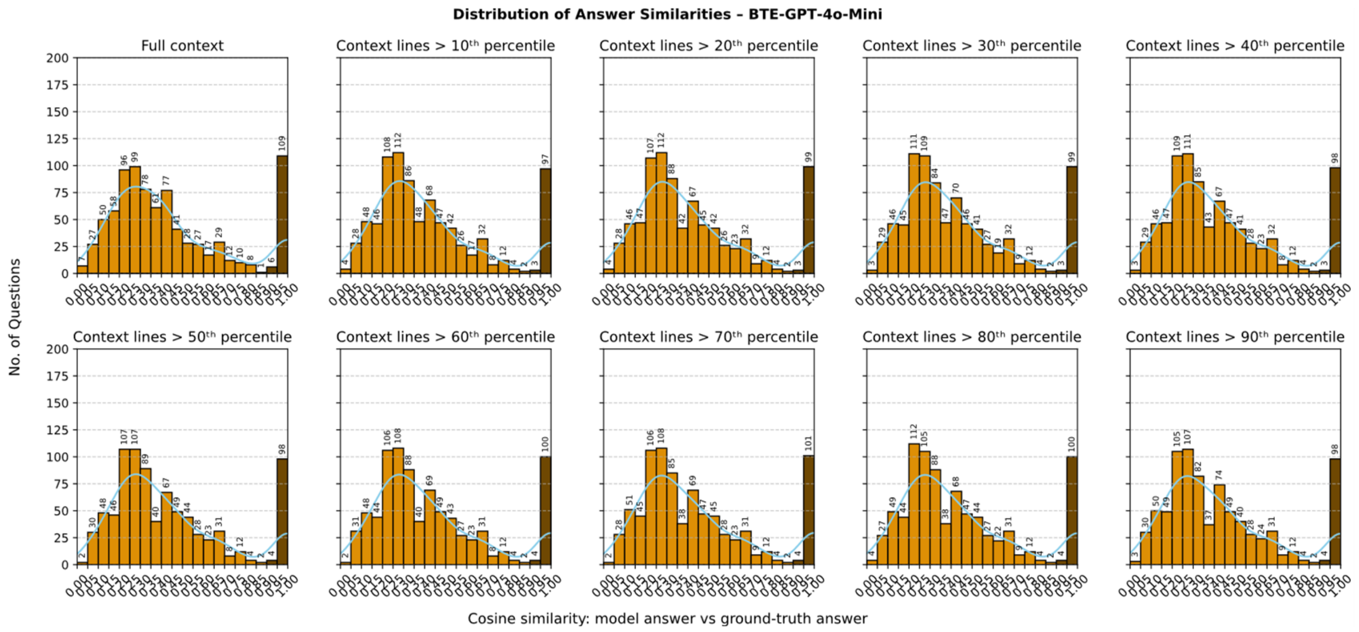

Figure S9: Distribution of answer similarities for the drug-centric benchmark using GPT-4o-mini in BTE-RAG mode.

Each panel shows the cosine similarity between model predictions and ground-truth answers when either the full retrieved context is used (top left) or when context lines are filtered above increasing cosine similarity percentiles (10th to 90th).

bioRxiv preprint doi: https://doi.org/10.1101/2025.08.01.668022; this version posted August 2, 2025. The copyright holder for this preprint (which was not certified by peer review) is the author/funder, who has granted bioRxiv a license to display the preprint in perpetuity. It is made available under aCC-BY 4.0 International license.

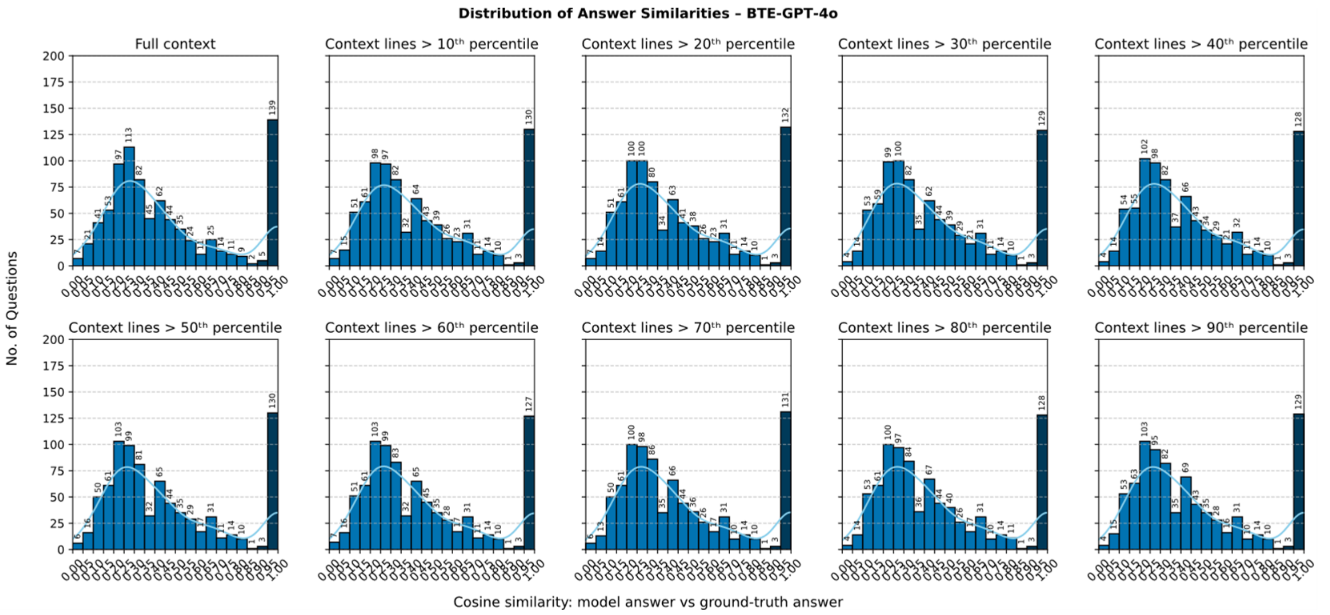

Figure S10: Distribution of answer similarities for the drug-centric benchmark using GPT-4o in BTE-RAG mode.

Each panel shows the cosine similarity between model predictions and ground-truth answers when either the full retrieved context is used (top left) or when context lines are filtered above increasing cosine similarity percentiles (10th to 90th).

bioRxiv preprint doi: https://doi.org/10.1101/2025.08.01.668022; this version posted August 2, 2025. The copyright holder for this preprint (which was not certified by peer review) is the author/funder, who has granted bioRxiv a license to display the preprint in perpetuity. It is made available under aCC-BY 4.0 International license.

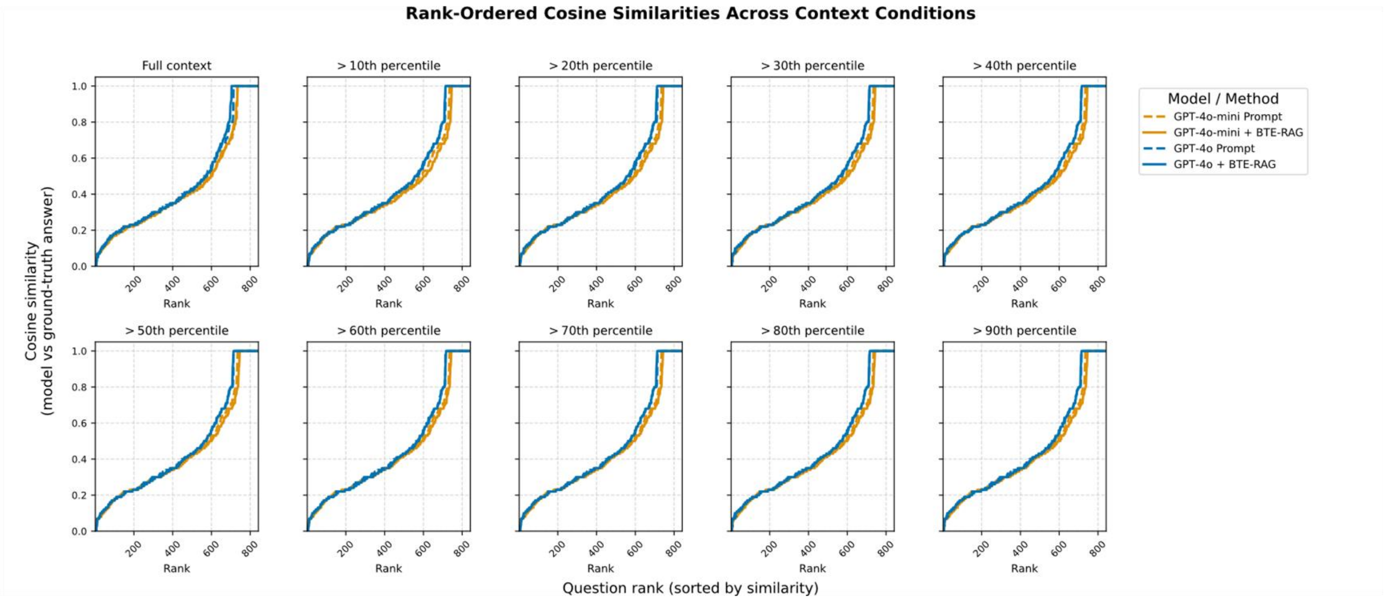

Figure S11: Rank-ordered cosine similarities between model predictions and ground-truth answers on the drug-centric benchmark, across context filtering thresholds.

Each panel displays results from four model–method combinations (GPT-4o-mini Prompt (LLM-only), GPT-4o-mini + BTE-RAG, GPT-4o Prompt, GPT-4o + BTE-RAG) under either full context or filtered context lines exceeding the indicated cosine similarity percentile (10th to 90th). Question predictions are sorted by similarity, revealing how context filtering and model selection affect semantic alignment with ground truth.

bioRxiv preprint doi: https://doi.org/10.1101/2025.08.01.668022; this version posted August 2, 2025. The copyright holder for this preprint (which was not certified by peer review) is the author/funder, who has granted bioRxiv a license to display the preprint in perpetuity. It is made available under aCC-BY 4.0 International license.

Table S1: System Prompts

Gene-centric benchmark

LLM-only	BTE-RAG
You are an expert biomedical researcher. Please provide your answer (only gene name) in the following JSON format for the Question asked:<br>{<br>"answer": <correct answer><br>}	You are an advanced biomedical researcher. Use your most recent knowledge in addition to the Context provided when needed to answer accurately.<br>*Answer Format*: Provide your answer (only the gene name) in the following JSON format:<br>{<br>"answer": <correct answer><br>}

Metabolite-centric benchmark

LLM-only	BTE-RAG
You are an expert biomedical researcher. Please provide your answer (only the biochemical entity name) in the following JSON format for the Question asked:<br>{{<br>"answer": <correct answer><br>}}	You are an advanced biochemistry researcher. Use your most recent knowledge in addition to the Context provided when needed to answer accurately.<br>*Answer Format*: Provide your answer (only the biochemical entity name) in the following JSON format:<br>{<br>"answer": <correct answer><br>}

Drug-centric benchmark

LLM-only	BTE-RAG
You are an expert biomedical researcher. Please provide your answer (only Drug names) in the following JSON format for the Question asked:<br>{{<br>"answer": <correct answer><br>}}	You are an **advanced biomedical research AI**, specializing in answering biological and biomedical questions with accuracy. Use the provided CONTEXT along with your most recent knowledge to answer the question.<br>*Answer Format*: Provide your answer (only Drug name) in the following JSON format:<br>{<br>"answer": <correct answer><br>}
